# Comparing Ultralong Carbon Nanotube Growth from Methane over Mono- and Bi-Metallic Iron Chloride Catalysts

**DOI:** 10.3390/nano13152172

**Published:** 2023-07-26

**Authors:** Tim Yick, Varun Shenoy Gangoli, Alvin Orbaek White

**Affiliations:** 1 Energy Safety Research Institute, Swansea University, Bay Campus, Swansea SA1 8EN, UK; 748963@swansea.ac.uk (T.Y.); v.s.gangoli@swansea.ac.uk (V.S.G.); 2 Department of Chemical Engineering, Faculty of Science and Engineering, Swansea University, Bay Campus, Fabian Way, Swansea SA1 8EN, UK; 3 TrimTabs Ltd., 63 St Christophers Ct, Swansea SA1 1UA, UK

**Keywords:** carbon nanotube, ultralong carbon nanotube, iron chloride, vapour-liquid-solid

## Abstract

This research endeavours to study the growth of ultralong carbon nanotubes (UL-CNTs) from methane using diverse catalysts, namely FeCl_3_, bi-metallic Fe-Cu, Fe-Ni, and Fe-Co chlorides. Aqueous catalyst solutions were evenly dispersed on silica substrates and grown at 950 °C in the presence of hydrogen via a horizontal chemical vapour deposition (CVD) furnace. The samples underwent characterisation by Raman spectroscopy, scanning electron microscopy (SEM), and optical microscopy to identify the quality of CNTs and enumerate individual UL-CNTs. Our findings revealed that FeCl_3_, as a mono-metallic catalyst, generated the longest UL-CNTs, which measured 1.32 cm, followed by Fe-Cu (0.85 cm), Fe-Co (0.7 cm), and Fe-Ni (0.6 cm), respectively. The G/D ratio (graphene to defects) from the Raman spectroscopy was the highest with the FeCl_3_ catalyst (3.09), followed by Fe-Cu (2.79), Fe-Co catalyst (2.13), and Fe-Ni (2.52). It indicates that the mono-iron-based catalyst also produces the highest purity CNTs. Moreover, this study scrutinises the vapour-liquid-solid (VLS) model for CNT growth and the impact of carbide formation as a precursor to CNT growth. Our research findings indicate that forming iron carbide (Fe_3_C) is a crucial transition phase for amorphous carbon transformation to CNTs. Notably, the iron catalyst generated the longest and densest CNTs relative to other iron-based bi-metallic catalysts, which is consistent with the temperature of carbide formation in the mono-metallic system. From correlations made using the phase diagram with carbon, we conclude that CNT growth is favoured because of increased carbon solubility within the mono-metallic catalyst compared to the bi-metallic catalysts.

## 1. Introduction

Potential applications of carbon nanotubes include producing polymer composites [1], supramolecular hydrogens [2], and electronics [3], such as batteries and electric wires. A critical bottleneck in using carbon nanotubes (CNTs) as long-range electron conductors is achieving type-specific growth of discrete chirality samples, particularly armchair chirality [4,5,6]. Until armchair-specific single-walled carbon nanotubes (SWCNTs) are readily available en masse, it is possible to create cables with high current densities using non-armchair CNTs so long as they exhibit a large aspect ratio [7], namely, CNTs with narrow diameters and ultralong lengths (UL-CNTs). UL-CNTs are typically formed using horizontal CVD systems employing methane and hydrogen with substrate-bound catalysts typically comprising mono-metallic formulations of iron [8], copper [9], cobalt [10], and nickel [11].

The growth of CNTs occurs through three general stages that follow: (i) carbon diffusion on the metal catalyst particle; (ii) carbon incorporation into the catalyst; and (iii) formation of a carbon nanotube structure upon carbon precipitation from the catalyst. Different metals can alter the rate of each reaction pathway. It has been shown that bi-metallic catalysts can increase the activation rate of the catalytic reaction, which in turn can assist carbon diffusion [12,13]. For example, copper addition to an iron chloride catalyst [14] achieved the highest growth activity over iron chloride. However, whether the bi-metallic catalysts can constantly improve the CNT growth rate over mono-metallic catalysts regardless of concentration and composition remain to be seen.

The use of wet chemistry techniques to prepare a catalyst is beneficial because the exact molarity of the material can be measured in advance so that the required concentrations can be easily prepared. However, a key challenge is the controlled dispersion of liquid catalysts so that one can relate the CNT growth to the dispersed catalyst concentrations. Catalyst concentrations are often prepared in organic solvents such as ethanol with a low surface tension [15]. However, when these solutions are applied to a cleaned Si/SiO_2_ surface, the catalyst solutions rapidly spread. Therefore, the final surface concentration is not linearly linked to the dispersed concentration, and thus it is challenging to relate the applied catalyst concentration to CNT growth. High surface tension media, such as deionised water (di-water), limit the spread of solutions on a surface and can therefore be used to control and contain the catalyst dispersion on a surface. Therefore, this controlled and confined region can help correlate CNT growth density with catalyst concentration.

Raman spectroscopy is useful to identify CNTs compared to fullerenes, graphene, or carbon fibres [16]. The spectrum of CNTs in Raman spectroscopy usually consists of a G-band (graphene band, ~1590 cm^−1^), a D-band (defects-induced band, ~1350 cm^−1^), and a 2D-band with a variable Raman shift (2600–2800 cm^−1^) [17]. Comparing the intensity of the G and D peaks indicates a qualitative value of CNT quality: a large G/D ratio indicates a smaller number of defects in the CNTs and represents higher- quality CNT material. The radial breathing mode (RBM) within 100–350 cm^−1^ is also a unique feature of single-walled carbon nanotubes (SWCNTs) due to the resonance of the singular tube structure [18].

Bi-metallic mixtures have been shown to have lower carbide formation temperatures, which leads to higher catalytic activities and enhances the growth rate of carbon nanotubes [19]. The melting point and carbide formation temperature of different catalysts, including iron (Fe), iron-copper (Fe-Cu), iron-nickel (Fe-Ni), and iron-cobalt (Fe-Co), have been evaluated in this study. The results showed that the mono-metallic catalyst (Fe) produced the longest carbon nanotubes with the highest quality. In contrast, Fe-based bi-metallic catalysts, such as Fe-Cu, Fe-Ni, and Fe-Co, produced shorter and lower quality carbon nanotubes. The Fe-Cu catalyst had the highest G/D ratio and density of carbon nanotubes, while the Fe-Co and Fe-Ni catalysts had the narrowest range of lengths and were typically shorter.

The fundamental principle of carbon nanotube growth can be explained using a vapour-liquid-solid (VLS) model [20]. The decomposition of solid carbon species from vaporised methane gases is dissolved on the surface of a liquid-phase catalyst. The precursor (carbon atoms) is continuously dissolved until saturated, which promotes carbon nanotube growth. Iron is commonly used as a catalyst because of its high catalytic efficiency [21]. The activation energy of the catalyst particle is equal to the diffusion energy of carbon atoms at the given temperature, which in this study was 950 °C. The adherent iron particles are mainly liquid throughout the heating process. Iron carbide (Fe_3_C) formation is a vital transition phase of amorphous carbon transformation into carbon nanotubes. The VLS model has been used to explain the growth of carbon nanotubes on various catalysts, including iron-based mono-metallic and bi-metallic catalysts. This study aims to provide valuable insights into carbon nanotubes’ growth rate and quality, which can be used to develop more efficient and effective catalysts for producing carbon nanotubes.

In this work, we used a water-diluted catalyst to disperse controlled quantities of catalyst onto Si/SiO_2_ surfaces to control catalyst location and concentration for UL-CNT growth. CNTs were grown from various catalyst systems and compared to a FeCl_3_ control sample using methane and hydrogen gases. This work further investigates whether the combination of transition metal catalysts could enhance the growth of UL-CNTs.

## 2. Materials and Methods

The types of catalytic substances used in the experiments: (1) Iron (II) chloride (FeCl_3_), (2) Copper (II) chloride dihydrate (CuCl_2_), (3) Cobalt (II) chloride (CoCl_2·_6H_2_O), and (4) Nickel (II) chloride hexahydrate (Cl_2_H_12_NiO_6_) were all used as received (Sigma-Aldrich, Dorset, UK). Samples of 10 mmol concentration in deionised-water (DI) were created by diluting weighed catalyst quantities of 16.2 mg (FeCl_3_), 17 mg (CuCl_2_), 13 mg (CoCl_2_·6H_2_O), and 23.8 mg (Cl_2_H_12_NiO_6_) into separate 20 mL vials and mixing via sonication for 10 min. Equimolar samples were created by combining appropriate FeCl_3_/water controls with the other transition metal salts individually, thus forming bi-metallic chloride samples. Before each growth reaction, the solutions were agitated using bath sonication to ensure homogeneous mixing.

Rectangular silicon wafer substrates (10 mm × 300 mm) and a silicon boat (18 mm × 500 mm) were cut from a 300 mm diameter N-type silicon disc (PI-KEM Ltd., Tamworth, UK) using a diamond scribe. The silicon wafers and silicon boat were cleaned using isopropyl alcohol in an ultrasonic cleaner for 10 min to remove any dirt and contaminants. The silicon wafers were then transferred to a glass dish using tweezers and dried on a 40 °C hot plate. Catalyst solutions were deposited on the silicon substrate using a Pasteur pipette approximately 1 mm from the leading edge of the silicon substrate.

CNTs were grown using a horizontal growth chemical vapour deposition (CVD) process inside a Carbolite Gero model 1200 tube furnace (Carbolite Gero, Sheffield, UK) using a quartz tube of 20 mm inner diameter and 1800 mm length (GPE Scientific Ltd., Bedfordshire, UK). The silicon boat was placed at the longitudinal centre line of the quartz tube, and the growth substrate was set 10 mm back from the leading edge of the boat. The placement of the boat and substrate was in the hottest zone of the furnace as measured using a thermocouple (see Supporting Information Figure S1), and a 950 °C growth temperature was used [22]. The feed gases of methane (low ethylene grade N3.5, CH_4_), helium (He), and hydrogen (H_2_) (BOC Ltd., Guildford, UK) were controlled by mass flow controllers (MFCs) (FMA-5400A, Omega Engineering, Manchester, UK) and regulated by pressure valves controlled by a Raspberry Pi 400 system running a custom Python script. The gas velocity of the system was regularly calibrated using a bubble meter to ensure accurate readings of the flow setpoint and flow output.

At the start of each growth reaction, the system is purged using helium (30 standard cubic centimetres per minute, SCCM) and hydrogen (30 SCCM) to remove any residual air and ensure a reducing atmosphere within the reaction tube. Upon initiation of the temperature ramp, He flow is set to 30 SCCM while H_2_ flow is ceased. Once the growth temperature of 950 °C is reached and maintained, the growth process begins by shifting the gas flow to methane (10 SCCM) and hydrogen (20 SCCM) for 15 min. After the growth duration, the samples are cooled to room temperature (~25 °C) under a He blanket (30 SCCM), following which the samples are removed for analysis.

The CNTs were characterised using resonant Raman spectroscopy and scanning electron microscopy (SEM). A Renishaw InVia Raman microscope and spectrograph (Pontyclun, UK) was used for qualitative analysis using three separate lasers of 633 nm (1.96 eV), 785 nm (1.58 eV), and 457 nm (2.71 eV) wavelengths with a 50× magnification lens. Beam power is throttled to maintain a high signal-to-noise ratio based on a 1% beam power for a 785 nm laser and a 5% laser power for 457 nm and 633 nm lasers; each sample spectrogram is acquired for a 25 s scan time with two accumulations. The samples are beam aligned by manoeuvring the sample z-height to achieve the greatest intensity of the G’ peak (~1650 cm^−1^) for the CNTs. Baseline correction and the removal of the 520 cm^−1^ silicon peak and any cosmic-ray peaks were carried out manually using the InVia WiRE (version 5.1) software to provide more details on the relevant carbon peaks.

SEM imaging was performed using a Jeol 7800F field emission scanning electron microscope (FE-SEM) (Tokyo, Japan) at 100× low magnification and aperture 3 with a 1 kV beam strength and scan speed set to maintain 1000 pA current density. Using the Low Electron Detector (LED) mode, the scan speed was set to 11 (approximately 100 μs dwell time), the image size was set to 5210 × 2140 pixels, and the working distance (WD) was 7 mm. The lower accelerating voltage can provide clarity and higher signal-to-noise images to help compensate for the rapid scan speeds and thus make the CNTs more visible [23]. Individual images were acquired to allow for the overlap of neighbouring images, which was often accounted for by using only 80% of each frame capture to be parsed onto a montage. Montages were created using GIMP software (Version 2.10).

## 3. Results and Discussion

**Figure 1:**
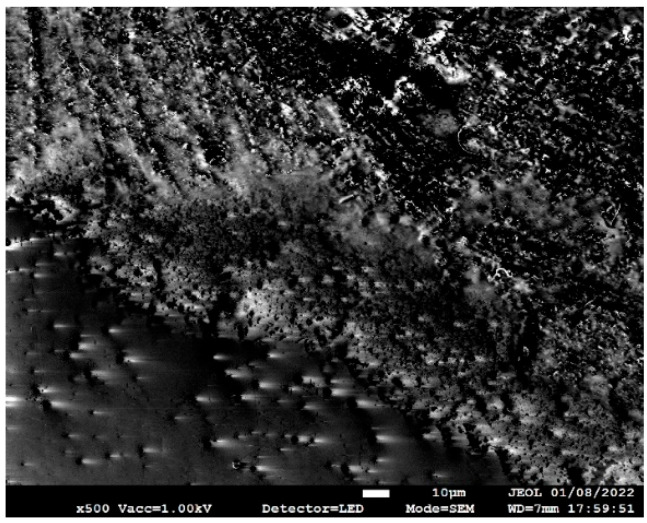
SEM image of the Fe-Cu catalyst in one area of the catalyst droplet at 100× magnification from the JEOL 7800F SEM.

To investigate the composition of the catalysts at an elevated temperature, the catalysts were individually dropped on a silicon wafer that was inserted into a quartz tube and heated up to 950 °C before being purged with helium gas at 100 SCCM. Figure 1 shows an example SEM image of a silicon wafer with a Fe-Cu catalyst droplet using the JEOL 7800F SEM at 100× magnification. The image reveals some unevenly distributed catalyst particles across the entire area of the catalyst droplet.

**Figure 2:**
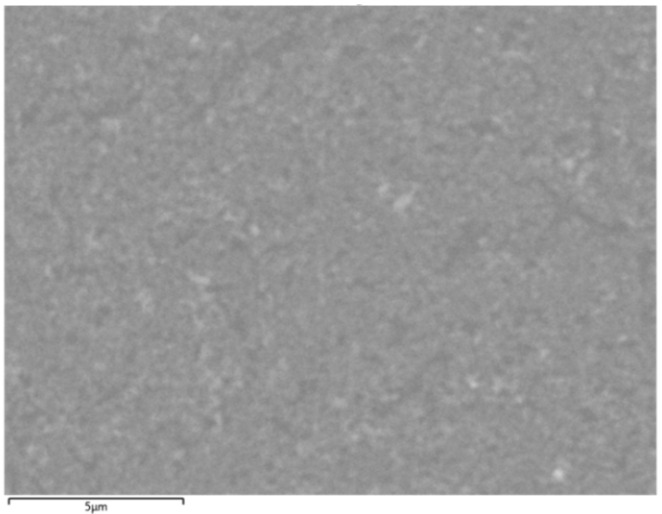
SEM image of a Fe-Cu catalyst from a Hitachi TM3030 SEM at 10,000× magnification.

To further confirm the composition of the catalysts (Fe, Fe-Cu, Fe-Co, and Fe-Ni), a tabletop scanning electron microscope (SEM) (Hitachi TM3030, Hitachi, Tokyo, Japan) with an integrated XSTREAM2 energy-dispersive X-ray spectroscopy (ESD) module (Oxford Instrument, Oxford, UK) was used. Figure 2 shows a zoomed-in section of the same Fe-Cu catalyst area captured above by the Hitachi TM3030 SEM at a 10,000× magnification for context.

**Figure 3:**
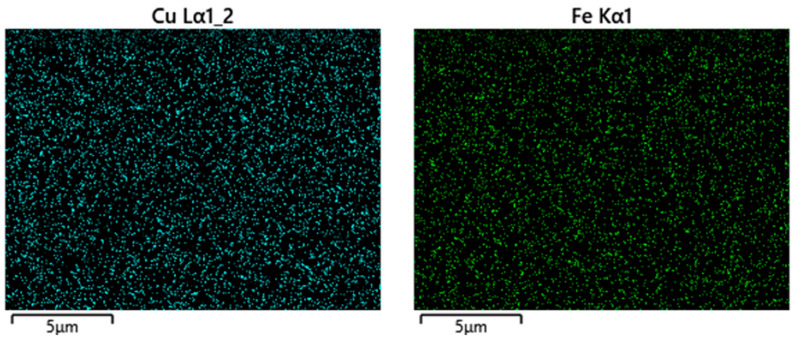
Fe-Cu catalyst composition maps for Cu (**left**) and Fe (**right**) using EDS analysis.

The working principle of energy-dispersive X-ray spectroscopy (EDS) is that the electron beam excites the sample and leads to X-ray emission due to vacant orbital formation, K-shell ionisation, and subsequent electron relaxation [24]. The different nature of this emission can be used to identify the atomic structure for chemical characterisations. For example, the distribution of copper (Cu) and iron (Fe) contents in the bi-metallic catalysts can be identified by this technique, as shown in Figure 3. Furthermore, the percentage of elements (%) can be determined by the ESD analysis. The iron (Fe), iron-copper (Fe-Cu), iron-cobalt (Fe-Co), and iron-nickel (Fe-Ni) catalyst samples were found to contain oxygen (O), silicon (Si), carbon (C), iron (Fe), and the corresponding elements (Cu, Co, and Fe), with weight percentage (%) and atomic percentage (%) (which is relative to the total number of atoms) listed in Table 1.

We see that most of the material on a weight percentage basis is silicon (Si) from the wafer on all four samples (Fe, Fe-Cu, Fe-Co, and Fe-Ni), which is not surprising given the nature of the samples, with small amounts of oxygen (O) and carbon (C) present. The FeCl_3_ control sample shows an Fe weight percentage of 8.18%, whereas the other bi-metallic samples have different ratios of composition, for example, Fe-Cu (Fe = 1.83%, Cu = 0.27%), Fe-Co (Fe = 1.62%, Co = 2.35%), and Fe-Ni (Fe = 1.06%, Ni = 3.47%). These experimental results indicate that the homogenous catalyst solutions are inconsistent with random compositions after deposition on the substrates. The mixture of bi-metallic catalysts and subsequent alloy formation are subject to complex crystallographic structures. It is further reported that the degree of alloy formation depends on the ratio of compositions and the amount of elements, with the temperature effects on the carbide formation and the melting temperature affecting the carbon nanotube growth [25,26].

**Figure 4:**
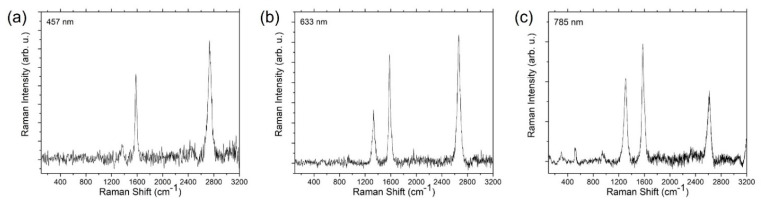
Raman spectroscopy data of CNTs grown from FeCl_3_ were measured using different lasers of (**a**) 457 nm, (**b**) 633 nm, and (**c**) 785 nm wavelengths.

**Figure 5:**
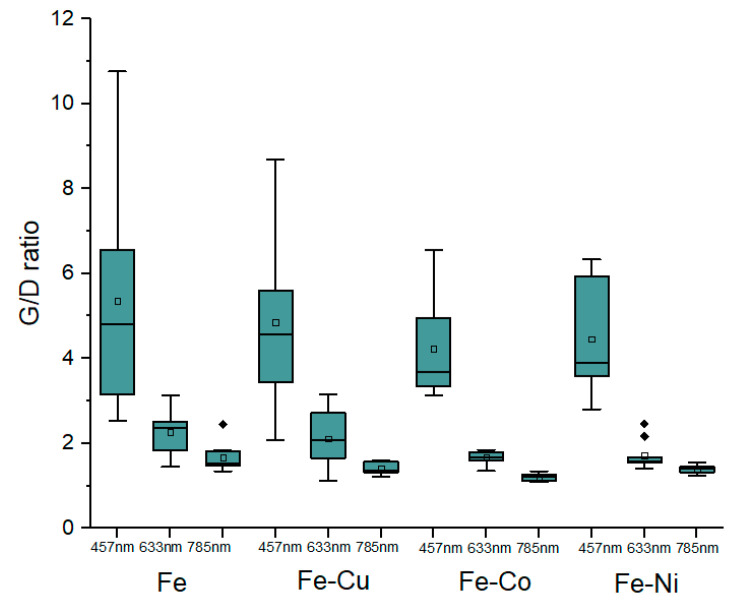
Box plot of G/D ratios for 457 nm, 633 nm, and 785 nm laser wavelengths.

Carbon nanotube characterisation has typically used Raman spectroscopy to identify the various carbon structures [27]. As seen in Figure 4, there was no evidence of radial breathing mode (RBM) peaks, suggesting these are not single-walled CNTs. The number of walls is unknown, so we report them merely as MWCNTs. The G-band (graphene band), D-band (defects-induced band), and a 2D-band with a variable Raman shift were present in the results collected at 457 nm, 633 nm, and 785 nm wavelengths. Occasionally, an iron oxide peak found at 223 cm^−1^ was detected in our samples. Multiple samples had strong silicon peaks found at ~520 cm^−1^, which is likely due to the sparsity of individual CNTs spread over a wide area of the silicon wafer, and this tends to wash out the signal intensity from the CNTs (hence the need to remove the Si peaks). The G/D ratios at 457 nm, 633 nm, and 785 nm wavelengths were obtained using five different locations across each sample to ensure statistical accuracy and are presented as a box plot in Figure 5.

**Figure 6:**
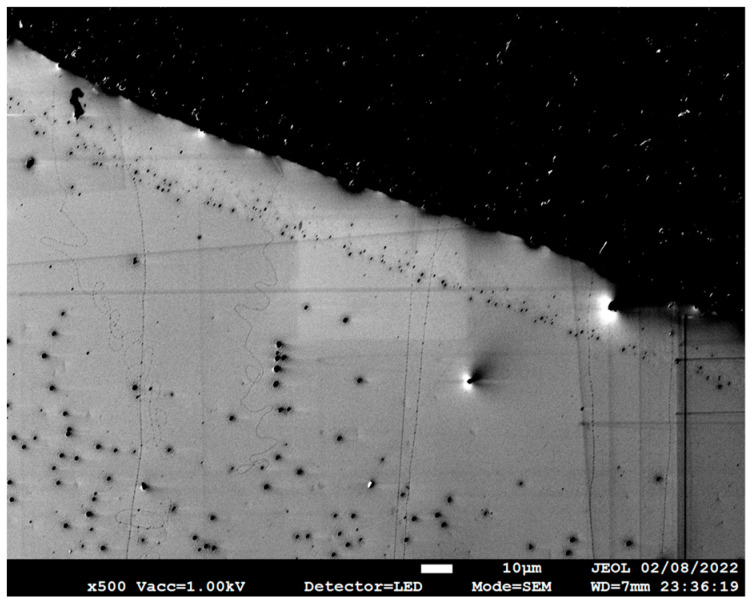
SEM image collected at 500× magnification near an FeCl_3_ catalyst droplet of CNTs grown from the catalyst.

**Figure 7:**
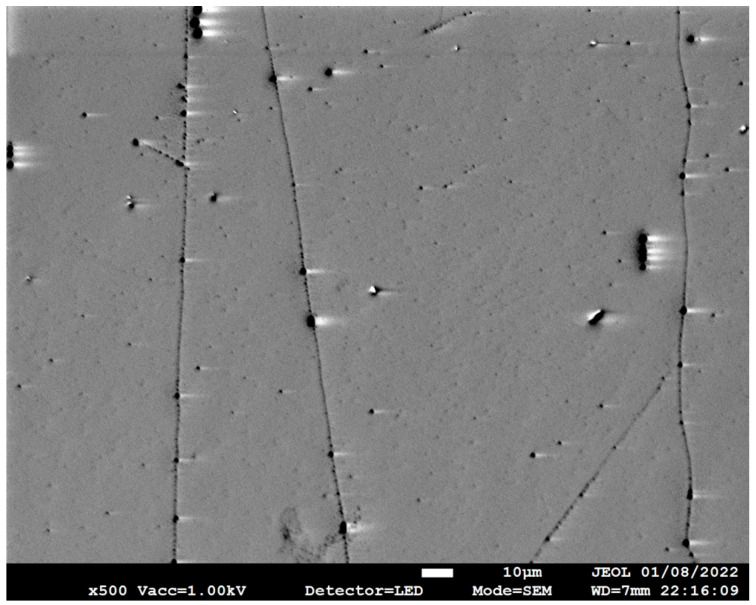
SEM image at 500× magnification of representative ultralong CNTs grown from FeCl_3_.

**Figure 8:**
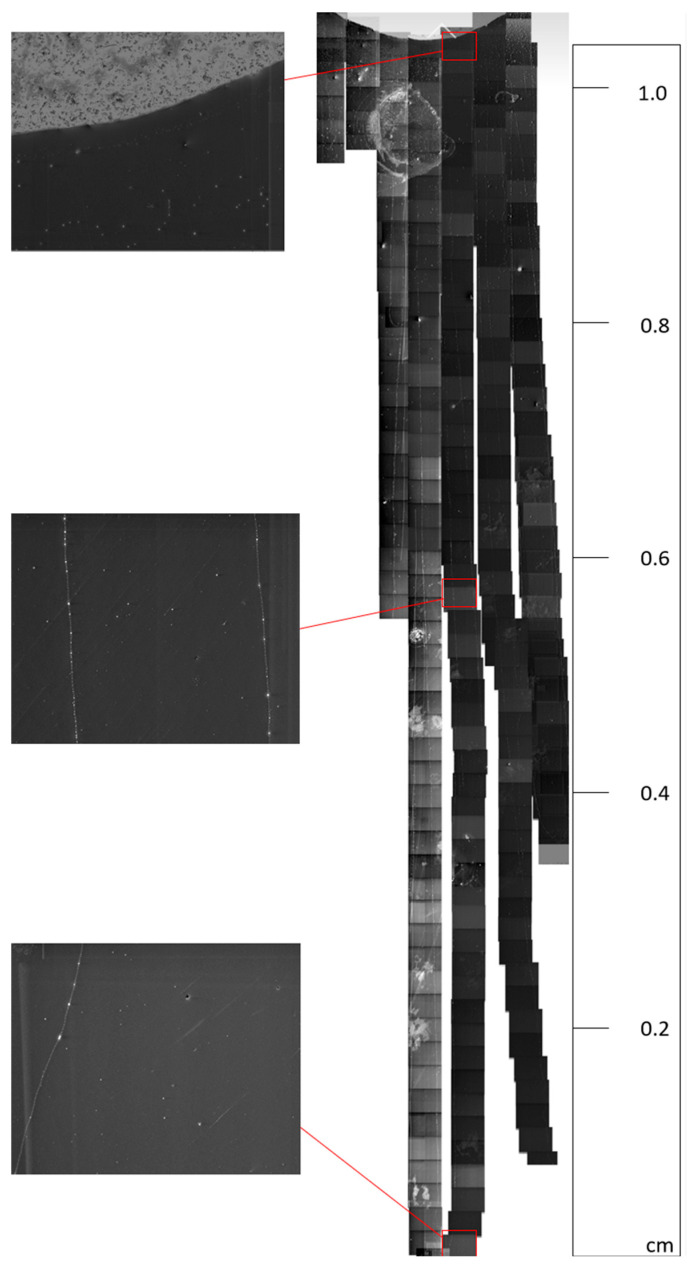
Stitched SEM images of example ultralong CNTs grown from FeCl_3_, with top, middle, and bottom images seen alongside for easier visualisation.

**Figure 9:**
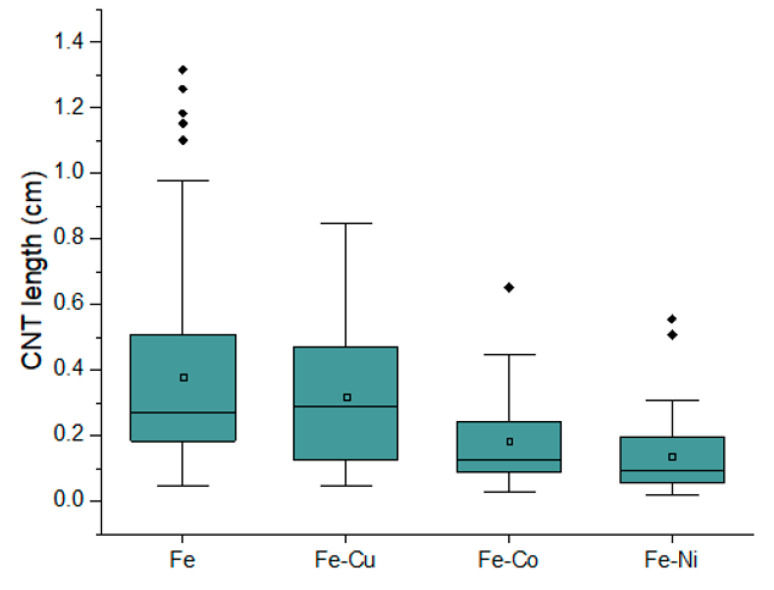
Box plot of CNT lengths grown from Fe, Fe-Cu, Fe-Co, and Fe-Ni catalysts.

The scanning electron microscope (SEM) images help visualise the grown CNTs and measure the CNT lengths using ImageJ [28]. Representative images in Figure 6, Figure 7 and Figure 8 show UL-CNTs grown from the FeCl_3_ control catalyst system. The catalyst islands (in Figure 6) show many short and curly carbon nanotubes, likely due to the catalyst concentrations being too high, thus lowering the probability of UL-CNTs being isolated from these islands due to CNT-CNT interactions. It is evident from the SEM images shown that using deionised water as a solvent to dissolve Fe, Fe-Cu, Fe-Co, and Fe-Ni catalysts in this work helped to contain the spread of catalyst on the Si surface. Given this restriction on how the catalyst droplet spreads, most of the UL-CNTs grow at the trailing edge of the droplet and follow the same direction as the gas flow. Table 2 lists the UL-CNTs, measured from longest to shortest. Our findings reveal that FeCl_3_, as a mono-metallic catalyst, generated the longest UL-CNT, measuring 1.32 cm, followed by Fe-Cu (0.85 cm), Fe-Co (0.65 cm), and Fe-Ni (0.55 cm), respectively. Box plots for all CNT lengths are shown in Figure 9.

Given that the growth duration lasted for 30 min and using the assumption that nanotubes grow consistently within that time frame, we can calculate a UL-CNT growth velocity of 0.73 µm/s, 0.47 µm/s, 0.36 µm/s, and 0.31 µm/s for Fe, Fe-Cu, Fe-Ni, and Fe-Co catalysts, respectively. The Fe catalyst has a broader range of CNT lengths (0.05–1.32 cm), followed by Fe-Cu (0.05–0.85 cm), Fe-Co (0.02–0.65 cm), and Fe-Ni (0.02–0.55 cm), with the latter also having typically shorter CNTs on average. The outliers in the Fe, Fe-Co, and Fe-Ni columns indicate that a few CNTs are notably longer than average and are the longest UL-CNTs. These data are also supported by the standard deviation (Table 2) of the highest iron chloride catalysts (0.23), Fe-Cu (0.16), Fe-Co (0.13), and Fe-Ni (0.12). The Fe catalyst displays more such outliers, and therefore, we suggest that the FeCl_3_ catalyst grows the longest carbon nanotubes with a higher probability of getting multiple UL-CNTs.

A Keyence VHX-7000 (Osaka, Japan) microscope was also used to determine the size of the catalyst islands. DI water enabled controlled catalyst deposition and allowed us to make logical assumptions about the fixed relationship between catalyst concentration and the density of UL-CNTs. The catalyst island areas ranged from 3.14 mm^2^ to 7.07 mm^2^, as noted in Table 3. The data were also visualised in terms of UL-CNT density per catalyst composition, showing that Fe resulted in the greatest number of ultralong CNTs.

The CNT quality can be determined by the highest G/D ratios and was found to change slightly depending on the laser wavelength. For the 457 nm laser, they were found to follow the order: Fe > Fe-Cu > Fe-Co > Fe-Ni; for the 633 nm laser, the order was Fe > Fe-Co > Fe-Cu > Fe-Ni, and for the 785 nm laser, it was Fe > Fe-Cu > Fe-Ni > Fe-Co.

The Fe catalyst has an average G/D ratio across all three wavelengths of 3.09, followed by the Fe-Cu catalyst at 2.79, the Fe-Co catalyst at 2.13, and the Fe-Ni system at 2.52. The standard deviation of the G/D ratios for all catalysts ranged from 0.86 to 3.2, with previous studies stating that measurements with a standard deviation of ±2 are acceptable [29]. Taking the average standard deviation for all categories, we calculated 2.04 for Fe, 1.88 for Fe-Cu, 1.62 for Fe-Co, and 1.44 for Fe-Ni. The Fe (as FeCl_3_) catalyst produces the widest range of G/D ratios compared to the other Fe-based bi-metallic catalysts. The maximum Raman intensity followed the same trend, with the Fe catalyst producing the best quality carbon nanotubes, followed by Fe-Cu, Fe-Co, and Fe-Ni. The descending order of the G/D ratio for a higher excitation wavelength of lasers, from 457 nm to 785 nm for each catalyst, as seen in Figure 5, is due to the incident light intensity [30]. Of the three lasers, namely the 457 nm (UV), 633 nm (visible), and 785 nm (near infrared) lasers, the 785 nm laser has a reduced scattering intensity compared to the other two given; generally, the Raman scattering intensity is proportional to the wavelength of the laser [31]. Another contributing factor is the spot size of the laser, which was 0.9 µm for the 514 nm laser and 1.7 µm for the 785 nm laser for the 20× optical objective [32] used, which may influence the data acquisition since the larger spot size has a greater area of interest within which to find the CNTs.

This study has shown that the mono-metallic catalyst (Fe) produces the greatest number of UL-CNTs, the longest UL-CNTs, and the CNTs with the highest G/D ratio compared to the bi-metallic catalysts. The principle of CNT growth can be explained using a vapour-liquid-solid (VLS) model [33]. Iron is commonly used because of its high catalytic efficiency [34] and activation energy matching the diffusion energy of carbon atoms at the operating temperature (950 °C) [35]. The adherent iron particles (Fe) are mainly in a liquid state throughout the heating process, and the melting point is lower than that of the bulk solid iron cluster. Co-coupling carbon atoms onto the molten catalyst particle’s surface would simultaneously promote longer carbon nanotube growth. The formation of iron carbide (Fe_3_C), which usually occurs at 1173–1100 K (900–1100 °C) [36], is a vital transition phase from amorphous carbon to high quality carbon nanotubes.

The Fe-Cu bi-metallic catalyst provided the next-best results in our study. A small quantity of Cu additive in a rich iron catalyst has been previously shown to promote longer CNT growth [37]. The miscibility of Cu particles into iron clusters leads to a stable condition to form carbon nanotubes, which plausibly explains the greater diameter control seen in our results. However, a higher concentration of Cu may end up hindering CNT growth [38]. Therefore, the Cu additive in equimolar concentrations is most likely to decrease the carbon solubility and consequently hurt UL-CNT growth compared to the results from the mono-metallic iron catalyst.

The Fe-Co catalyst was the third-most active catalyst system in this study. The typical CNT growth temperature with Fe-Co is 650–800 °C [39], which logically means there is reduced catalytic performance when the growth temperature is 950 °C. The shorter CNTs may be attributed to the carbon forming additional CNT walls in this constrained carbon diffusion system rather than growing longer CNTs.

The narrow range of CNT length for the Fe-Ni catalyst in this study matches previously reported results [40]. It is commonly known that an active transition metal for graphitisation reactions, which lowers CNT quality, may be due to excessive graphitic formation at the growth temperature [41]. Given that the Fe-Ni catalyst resulted in the lowest G/D ratios and the shortest average CNT length, it is postulated that the activation energy for Fe-Ni to grow CNTs is relatively high at this elevated temperature, resulting in a lowered carbon diffusion rate through the catalyst. The competition for growing longer CNTs rather than additional walls is similar to the iron-cobalt (Fe-Co) system.

## 4. Conclusions

In conclusion, the iron catalyst had the highest activity among the various catalysts studied for increasing the probability of ultralong CNT growth. The mono-metallic Fe catalysts resulted in the longest nanotubes (1.32 cm), followed by the Fe-Cu (0.85 cm), Fe-Co (0.65 cm), and Fe-Ni (0.55 cm) catalysts. The Fe catalyst also has the most UL-CNTs per catalyst area (17.69, 9.64, and 7.67 for the three samples studied), resulting in the densest UL-CNTs compared to the other catalysts. The mono-metallic formulation using FeCl_3_ had the broadest range of G/D values, as seen from the maximum Raman G/D intensity (7.85, 4, and 10.76 for the three samples studied), suggesting the quality of the CNTs varied considerably compared to the other formulations. The mono-metallic iron catalysts produced both the longest and highest quality CNTs, compared to other iron-based bi-metallic catalysts, such as iron-copper (Fe-Cu), iron-nickel (Fe-Ni), and iron-cobalt (Fe-Co). The vapour-liquid-solid method (VLS model) has been used to explain the principle of CNT growth, where the precursor (carbon atoms) is dissolved onto the surface of a liquid-phase catalyst, thus promoting carbon nanotube growth. The formation of iron carbide (Fe_3_C) is also important for transforming amorphous carbon into carbon nanotubes, with the highest maximum G/D ratio observed where carbide formation is promoted. This study provides valuable insights towards optimising CNT growth for the preferential production of ultra-long CNTs, which have many potential applications, including electric wires and telecommunication [42].

## Figures and Tables

**Table 1:** Weight and atomic percentage in the map sum spectrum.

	Fe		Fe-Cu		Fe-Co		Fe-Ni	
Map Sum Spectrum	Weight %	Atomic %	Weight %	Atomic %	Weight %	Atomic %	Weight %	Atomic %
O	8.14	12.93	10.36	15.85	6.79	10.37	7.25	10.37
Si	87.14	78.90	81.58	71.10	85.50	60.15	73.78	60.15
C	3.62	7.67	5.96	12.14	3.74	27.52	14.44	27.52
Fe	8.18	12.94	1.83	0.80	1.62	0.43	1.06	0.43
Cu	-	-	0.27	0.11	-	-	-	-
Co	-	-	-	-	2.35	1.09	-	-
Ni	-	-	-	-	-	-	3.47	1.52
Total	100.00	100.00	100.00	100.00	100.00	100.00	100.00	100.00

**Table 2:** The longest CNT lengths and the calculated growth rate from the various catalysts.

Chloride Catalyst	Longest CNT Observed (mm)	Average CNT Length (mm)	Standard Deviation (mm)	Calculated Growth Velocity (μm/s)
Fe	1.32	0.42	0.23	7.33
Fe-Cu	0.85	0.36	0.16	4.72
Fe-Co	0.65	0.20	0.13	3.61
Fe-Ni	0.55	0.14	0.12	3.06

**Table 3:** Catalyst diameter, CNT count, and density from catalyst islands and Raman G/D values.

Type of Catalyst	Sample No.	Catalyst Area (mm^2^)	CNT Count	Density (CNT per mm^2^)	Average Raman G/D Intensity	Standard Deviation Raman G/D Intensity	Maximum Raman G/D Intensity
Fe	Sample 1	5.31	46	8.66	3.10	2.07	7.85
	Sample 2	6.16	27	4.38	2.33	0.86	4.00
	Sample 3	7.07	23	3.25	3.85	3.20	10.76
Fe-Cu	Sample 4	3.80	19	5.00	2.21	1.10	4.57
	Sample 5	5.31	12	2.26	3.10	2.87	4.32
	Sample 6	4.15	20	4.82	3.06	1.68	5.59
Fe-Co	Sample 7	5.31	23	4.33	2.09	1.11	3.68
	Sample 8	6.16	11	1.79	2.14	1.69	4.18
	Sample 9	3.80	16	4.21	2.17	2.06	3.67
Fe-Ni	Sample 10	3.14	9	2.87	2.41	1.05	4.32
	Sample 11	6.16	11	1.79	2.36	1.11	6.33
	Sample 12	3.80	15	3.95	2.79	2.16	6.32

## Data Availability

Data is unavailable due to privacy restrictions.

## Supplementary Materials

The following supporting information can be downloaded at: https://www.mdpi.com/article/10.3390/nano13152172/s1, Figure S1: Temperature distribution in Carbolite Gero model 1200 tube furnace.
