# Supplementary material for: Comparing Ultralong Carbon Nanotube Growth from Methane over Mono- and Bi-Metallic Iron Chloride Catalysts
Source: Nanomaterials (Basel). 2023 Jul 26;13(15):2172. doi: 10.3390/nano13152172 (PMC10421160; doi:10.3390/nano13152172)
Supplement: Supplementary file 1 [file nanomaterials-13-02172-s001.zip › nanomaterials-2420014-supplementary.pdf]

Comparing Ultralong Carbon Nanotube Growth from Methane over Mono- and Bi-Metallic Iron Chloride Catalysts

Tim Yick[1], Varun Shenoy Gangoli[1,2], and Alvin Orbaek White[1,2*]

Supporting information

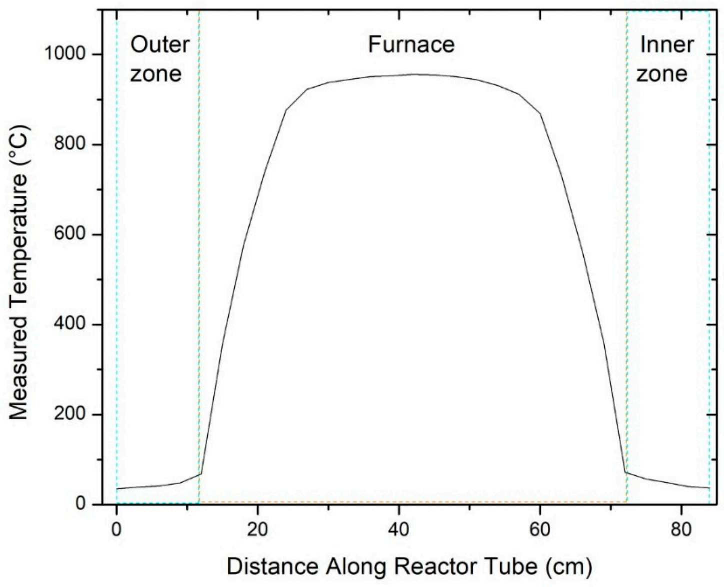

Figure S1. Temperature distribution in Carbolite Gero model 1200 tube furnace (Carbolite gero, Sheffield, UK)
